# Capturing Natural-Colour 3D Models of Insects for Species Discovery and Diagnostics

**DOI:** 10.1371/journal.pone.0094346

**Published:** 2014-04-23

**Authors:** Chuong V. Nguyen, David R. Lovell, Matt Adcock, John La Salle

**Affiliations:** 1 Computational Informatics, Commonwealth Scientific and Industrial Research Organization (CSIRO), Canberra, ACT, Australia; 2 Ecosystem Sciences, Commonwealth Scientific and Industrial Research Organization (CSIRO), Canberra, ACT, Australia; 3 Atlas of Living Australia, Canberra, ACT, Australia; Institut National de la Recherche Agronomique (INRA), France

## Abstract

Collections of biological specimens are fundamental to scientific understanding and characterization of natural diversity—past, present and future. This paper presents a system for liberating useful information from physical collections by bringing specimens into the digital domain so they can be more readily shared, analyzed, annotated and compared. It focuses on insects and is strongly motivated by the desire to accelerate and augment current practices in insect taxonomy which predominantly use text, 2D diagrams and images to describe and characterize species. While these traditional kinds of descriptions are informative and useful, they cannot cover insect specimens “from all angles” and precious specimens are still exchanged between researchers and collections for this reason. Furthermore, insects can be complex in structure and pose many challenges to computer vision systems. We present a new prototype for a practical, cost-effective system of off-the-shelf components to acquire natural-colour 3D models of insects from around 3 mm to 30 mm in length. (“Natural-colour” is used to contrast with “false-colour”, i.e., colour generated from, or applied to, gray-scale data post-acquisition.) Colour images are captured from different angles and focal depths using a digital single lens reflex (DSLR) camera rig and two-axis turntable. These 2D images are processed into 3D reconstructions using software based on a visual hull algorithm. The resulting models are compact (around 10 megabytes), afford excellent optical resolution, and can be readily embedded into documents and web pages, as well as viewed on mobile devices. The system is portable, safe, relatively affordable, and complements the sort of volumetric data that can be acquired by computed tomography. This system provides a new way to augment the description and documentation of insect species holotypes, reducing the need to handle or ship specimens. It opens up new opportunities to collect data for research, education, art, entertainment, biodiversity assessment and biosecurity control.

## Introduction

Technology has a critical role to play in accelerating the understanding of biological diversity and, for decades, scientists have strived to create accurate 3D duplicates of plants and animal specimens [1]. This paper describes a novel method of using technology to liberate information about physical specimens by bringing them into the digital domain as natural-colour 3D models—consistent with ideas and directions articulated by several other authors [2]–[10]. In particular, the proof of concept system we present fits well with the suggestion of Wheeler *et al*. [11] to “engineer and deploy a network of automated instruments capable of rapidly creating 3D images of type specimens” as part of a larger strategy of dealing with the massive backlog of insect types that are not yet digitized in any form. High resolution 3D scans, as well as being useful as versatile replicas, also have the potential to act as a common frame of reference for other data relating to the original insect such as annotations, auxiliary image collections, and measurements. These additional aspects are vital for the ways taxonomists convey the various morphological characters that distinguish a new species from those previously discovered.

Our work is focused on the digitization of insect species, building on research and development at the Australian National Insect Collection (ANIC) which currently holds over 12 million specimens, and is growing by around 100,000 specimens every year. Our mission is to enable high-quality 3D models of insects to be acquired quickly and cheaply, for ANIC to use as a component of its digitization strategy. Like many Natural History collections around the globe, the ANIC maintains many (thousands) Holotypes - each the single specimen of a species that is used to define the characteristic features of that species. Holotypes exist as a physical object carefully protected from damage through handling. Digital colour 3D models of sufficient detail will enable collections managers to liberate these precious specimens for the research work they are intended to fulfill.

Micro Computed Tomography (Micro CT) is currently a key method [12], [13], able to create micron-accurate volumetric models of millimeter-scale objects and their internal structure. However, like recent 3D reconstructions from scanning electron microscope (SEM) micrographs [14], [15], Micro CT is unable to capture important information about the surface of the object: its natural colour. Exposure and reconstruction times can be long (tens of hours) and, as an X-ray imaging method, Micro CT generally demands special safety equipment. Current systems cost in the hundred-thousand dollar range and, while more compact desktop models are available, these are still not especially portable.

The inability of X-ray based methods for insect digitization to capture colour led us to consider image-based 3D reconstruction techniques as reviewed in [16], [17]. These methods have been successfully applied to the reconstruction of 3D cityscapes and other (generally fairly simple) objects [18]–[20]. Some small biological specimens have been digitized [21]–[23] but the methods used do not specifically cater for the complex structures and challenging surface optical properties of insects. Human-in-the-loop approaches have been proposed for insect modeling [24] as have methods (limited to simple insect geometries) for inferring 3D insect shape from a single 2D image [25]. Experiments [26], [27] with laser scanning systems like [28] have suggested that this approach has difficulties with the fine structures and the small scale of many insects, as well as reflective, transparent or iridescent surfaces.

One way to avoid these difficulties is to steer clear of 3D reconstruction altogether and simply present 2D images obtained from different viewing angles [29]. While this method of 3D visualization is popular for museum collections it does not provide the quantitative information (e.g., 3D morphology) needed to analyze and compare insect specimens. Furthermore large amounts of data are involved: many high-resolution images are needed to give a convincing illusion of looking at an actual 3D object. This makes smooth, realistic interaction difficult and precludes straightforward email exchange or embedding of the object data.

In summary, there is a lack of existing systems that could capture the 3D structure and surface optical properties of small, intricate insect specimens at sufficient resolution for ANIC and other collections to digitize, share, analyze and compare their holdings. The rest of the paper describes our prototype system and its operation, and how it has achieved these design objectives.

## Materials and Methods

**Figure 1:**
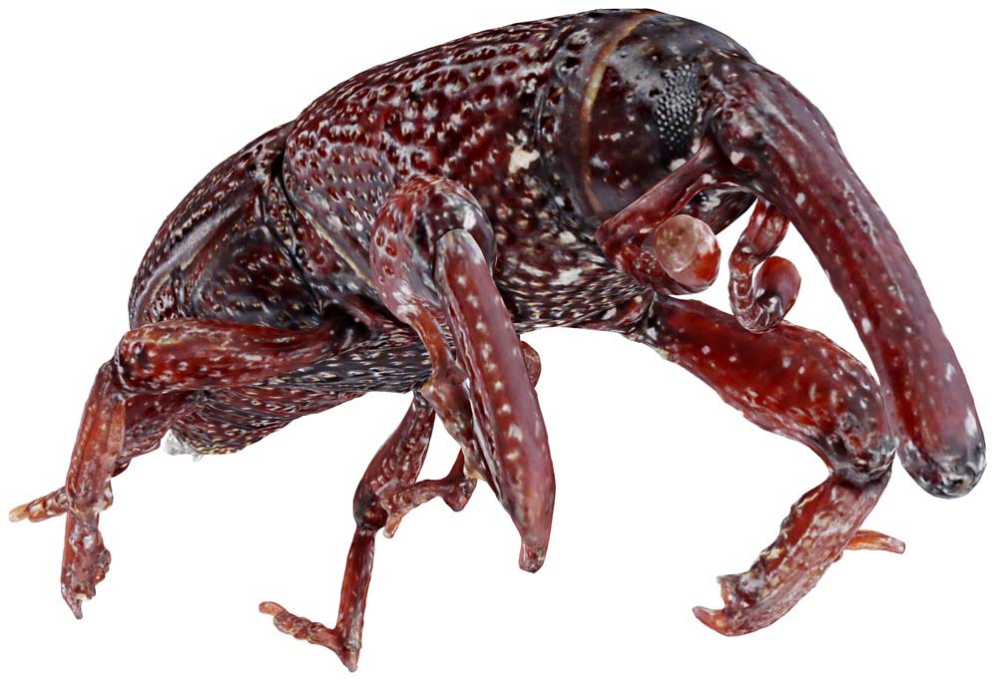
3D visualisation of a granary weevil on web as part of a video showing an overview of the 3D scanning process. Go the link at [30] to view the video.

Here we provide overviews of the digitization process and equipment. A video [30] as depicted in Figure 1 shows the main components of the system and the digitization process in action.

### Process overview

**Figure 2:**
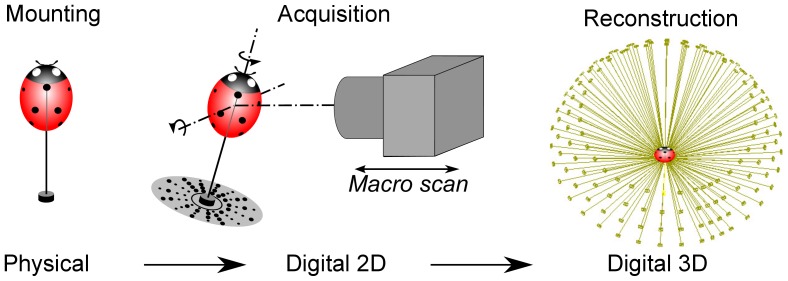
The three main steps to create a natural-colour 3D model of specimen. The steps are mounting the insect onto a pin, acquisition of 2D images of the specimen at different poses, then reconstruction of a single 3D model from those multiple images.

In high-level terms, our system and work-flow involve three main steps (Figure 2):

**Mounting**. the physical specimen is pinned onto a pre-printed mat used later by the reconstruction software to estimate camera pose (viewing angle and position).

**Acquisition**. 2D images of the specimen are automatically acquired from different orientations (and focal depths for small insects). This step marks the transition from the physical to the digital domain.

**Reconstruction**. in which a 3D model is inferred from multiple 2D images. For small insects, this involves multi-focus image stacking before the general steps of extracting camera pose, shape and colour.

The system has two modes of acquisition, depending on the specimen size. Insects larger than 10 mm are captured in *normal-mode* in which the depth of focus of the normal DSLR camera lens is enough to keep the whole specimen in focus at any viewing angle. Insects smaller than 10 mm are captured in *macro-mode* using a high-magnification lens. Because of the shallow depth of focus of this lens, multiple images are captured at different distances from the specimen and processed into a single in-focus image.

### Equipment overview

**Figure 3:**
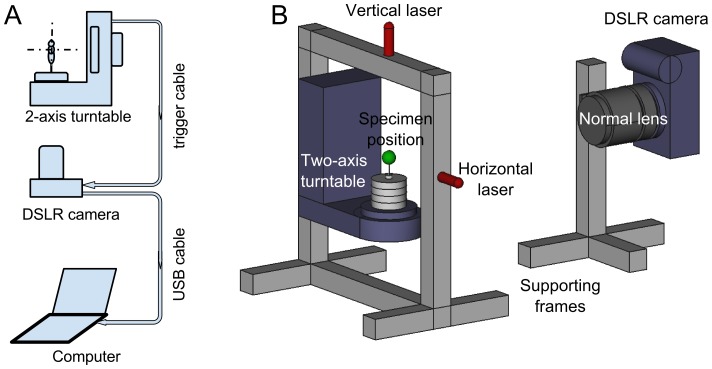
Connections (A) and hardware (B) for *normal-mode* image acquisition. The green sphere marks the center of rotation and mounting location of specimens. The turntable is the master device that triggers the camera after rotating to predetermined pan and tilt angles. Images can be stored in camera memory or transferred directly to the computer as they are acquired.

**Figure 4:**
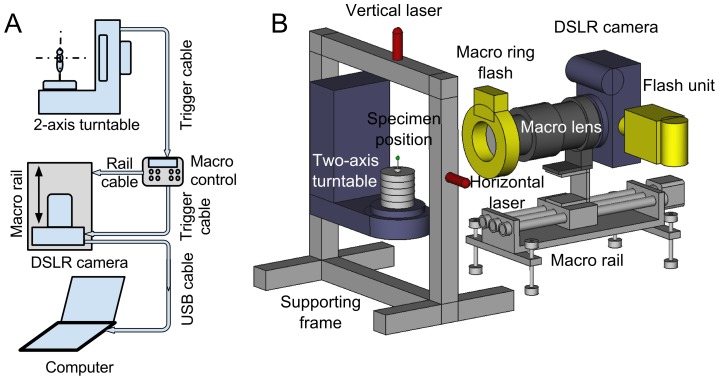
Connections (A) and hardware (B) for *macro-mode* image acquisition. The macro lens, macro ring flash and macro-rail are needed for capturing high-magnification and depth-extended images of small insects. At each rotation step, the turntable triggers the control box of macro-rail. The macro-rail then moves to a set of predetermined positions. At each position, the control box triggers the camera to capture an image.

Figures 3 and 4 show normal- and macro-mode setups. The main hardware components of the system are:

- A two-axis turntable to present views of the specimen from different angles of rotation
- A macro-rail to vary the distance between the camera and specimen in macro-mode
- A camera with macro lens and flash.
- Two laser pointers for specimen alignment
- A computer for 2D image processing and 3D reconstruction.

It is noted that in macro-mode our system uses a macro-rail to capture multi-focus images exactly at predefined depths, as opposed to refocusing the camera lens. A camera flash is needed to eliminate motion blur due to camera shutter's vibration when capturing at high magnification.

To minimize cost and development time we sought to use off-the-shelf components wherever practicable. These are described in detail in Supplementary Information S1.

### Process in detail

#### Step 1: Mounting

Collections usually store and display insects larger than 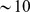
mm by pinning them so that the insect's long axis is horizontal and the pin vertical. Insects smaller than 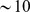
mm are usually either pinned or glued in cards. This paper however focuses on pinned insects and issues arising from this mounting method. Pinning insects horizontally allows many insects to be stored in wide, flat display drawers but creates a few problems for our system:

- The pin becomes part of the 3D model and must be edited or segmented out in post-reconstruction
- Editing can often not fully remove evidence of the pin
- Images of the underside of the specimen can be difficult or impossible to capture, leading to an incomplete 3D model.

Re-pinning the insect so its long axis is vertical helps with image acquisition but risks damaging the specimen, including parts, such as genitalia, that are important for the identification of some species. For some specimens, these affected parts can be isolated through dissection and scanned separately.

**Figure 5:**
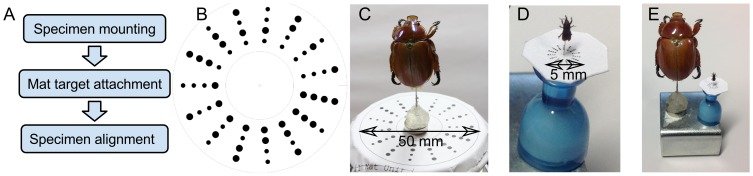
Preparing insect specimen for scanning. A) Steps to prepare insect specimens for image capturing. B) A special mat target needs to be attached to a scanned specimen for 3DSOM software to estimate of camera viewing position and angle. C) For a large insect such as this 30 mm long Christmas beetle, the pin is glued to a 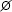
10 mm rare-earth disk magnet which is in turn attached to a 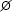
50 mm mat target. D) For a small insect such as this 3 mm long granary weevil, the micro pin is glued to a 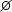
5 mm mat target. E) shows comparison in size of the two specimens.

After the specimen is pinned, the pin is glued to a small magnet (Figure 5C) that will hold the pin in position on the turntable. Next, a specially patterned mat (Figure 5B), required by the reconstruction software (3DSOM [31]), is attached to provide information about camera pose and position relative to the specimen. Generally the suitable size of the pattern is about one to two times the length of the insect to be scanned. Scanning smaller insects requires smaller patterns to be printed. Currently, modern laser printers with 1200 dpi printing resolution can produce patterned mats as small as 5 mm in diameter. Printing smaller patterns that are sharp enough to be recognised by the reconstruction software is currently a technical challenge.

Finally, the whole assembly is placed on the two-axis turntable and positioned (with the assistance of horizontal and vertical laser pointers) so the specimen is centered on the intersection of the axes of tilt and rotation. The lasers are aligned to the rotation axes of the turntable. A specimen is manually aligned to each of the laser beams such that each beam hits the centre of the insect's body.

#### Step 2: Acquisition

**Figure 6:**
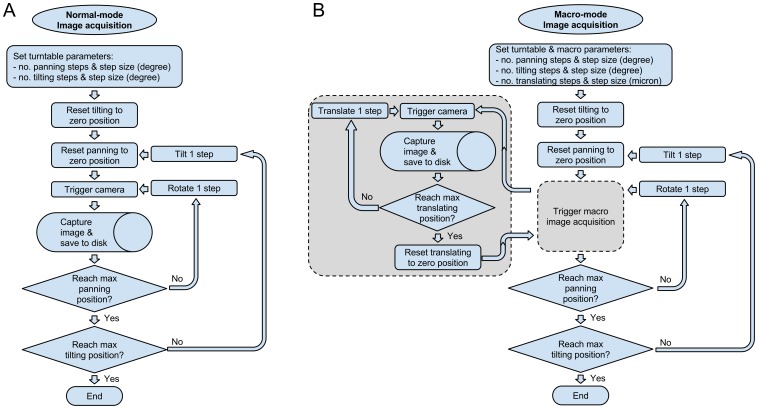
Automated image acquisition process. A) Normal-mode. B) Macro-mode.

In essence, the acquisition process is about automatically obtaining 2D images of the specimen in different poses. As far as the relationship between the camera and specimen goes, this system has three degrees of freedom: pan, tilt and (in macro-mode) distance along the specimen-camera axis. With the specimen mounted at the intersection of the pan and tilt axes of the turntable, this amounts to rotating the turntable through a range of pan and tilt angles, capturing an image at each step (Figure 6A). In macro-mode there is an additional “inner loop” of translating the camera to acquire partially focused images at different distances from the specimen for later processing into a single image with all parts of the specimen fully in focus (Figure 6B).

There are many ways to automate the acquisition process. The desire to use off-the-shelf components led us to use the GigaPan Panorama Robot EPIC 100 [32] for mounting the *specimen*. The GigaPan is designed for mounting and controlling a *camera*—and this led to the GigaPan robot also acting as the acquisition controller. In other words, it is the turntable that triggers the macro-rail. The macro-rail moves and triggers the camera which triggers its flash and takes an image. Supplementary Information S1 contains more detail about this set-up.

In normal-mode, using rotation and axis tilt, the set-up captures 144 individual images. In macro-mode, the additional up to 31 images required at each step mean that the system can capture up to 4,464 separate images per specimen. Capturing more images is also possible.

#### Step 3: Reconstruction

**Figure 7:**
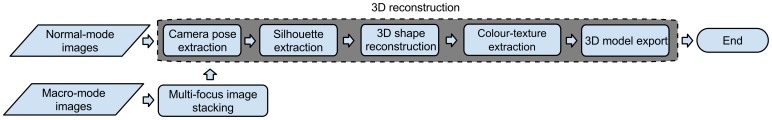
Image processing pipeline for normal-mode and macro-mode images. Macro-mode images require an extra step to stack each set of multi-focus images captured from the same viewing angle (but at different depth distances) into a single in-focus image.

The third and final step of the digitization process is where the 2D digital information acquired from a physical specimen is manipulated to produce a 3D digital model (Figure 7).

In macro-mode, the stack of partially focused images acquired at different specimen-camera distances must be combined into a single in-focus image for a given viewing angle. We used Helicon Focus [33] for this because of its ability to exploit multiple CPU cores. Single core open-source alternatives are available [34], [35].

Armed with a set of in-focus 2D images of an object from different viewing angles, there are two main 3D reconstruction techniques that could be applied:

**Visual hull** (also known as *volume carving*) algorithms [36], [37] project the silhouette of the object into a virtual volume at each viewing angle, carving away the volume outside the silhouette to leave a 3D visual hull which approximates the shape of the actual object. This approach does not recover concave surfaces, but photo-consistency can be used to correct this to an extent[38]. The extent of improvement by photo-consistency is limited for some insects due to strong speculiar reflections on the outer-surface and fine body structures such as legs, antennae, spikes and hairs.

**Multi-view stereo** algorithms generally rely on photo-consistency measures to identify the location of common features seen in different views [39], [40] and can also incorporate silhouette information [41].

Both strategies are computationally intensive and the computational demands increase with reconstruction resolution. Image clustering [18], [42] and improved feature descriptors [20] have been previously proposed to enable reconstructions to better exploit the very high image resolution produced by professional photography cameras.

Our initial investigations indicated that the visual-hull-based method could more accurately reconstruct some of the thin structures found in insects (e.g., legs, antennae, wings) and insect surfaces with strong specular reflections. 3DSOM [31] was used to provide off-the-shelf visual-hull-based reconstruction as it produced the best quality output of the different approaches [42]–[44].

Figure 7 sets out the detail of the reconstruction process, including the extraction of the camera pose in each input image. 3DSOM initially estimates this information from the target pattern captured in the image and further refines these estimates during 3D reconstruction. Specimen silhouettes are extracted from input images. Once the 3D geometry of the specimen's surface is reconstructed, texture colour is extracted from the images and added to the model. The resulting 3D model can be then exported to different formats—including HTML (with WebGL, Flash or Java), X3D, 3DS (AutoDesk), and STL (STereoLithography)—for subsequent viewing, analysis or embedding into documents. X3D is a convenient format as it is supported by popular 3D visualisation software, and a X3D file can included as an embedded object or as XML inline in an HTML5 file for 3D web visualisation. InstantReality's [45] tool “aopt” can perform this conversion X3D to 3D-supported HTML automatically.

## Results and Discussion

**Figure 8:**
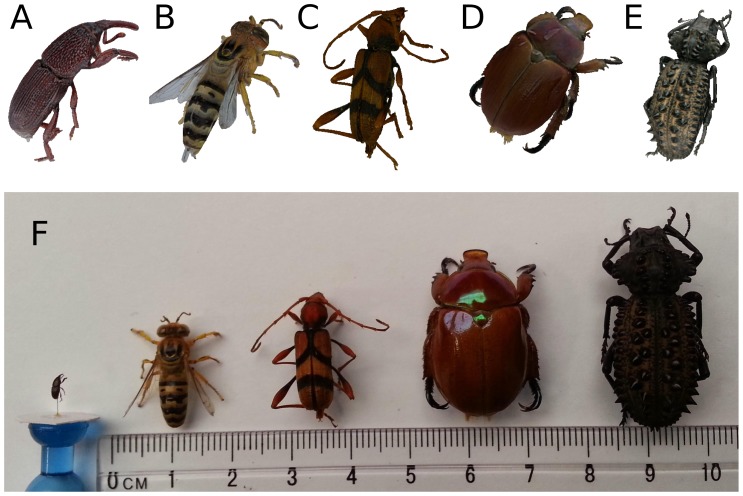
Various 3D insect models. Go to the link at [46] to interact with the 3D models or to the links at [47]–[54] to download. Top: 3D models of the insects with natural-colour texture, scaled to have similar sizes. They are A) a granary weevil (*Sitophilus granarius*), B) a sand wasp (*Bembix sp*.), C) a longhorn beetle (*Aridaeus thoracicus*), D) a Christmas beetle (*Anoplognathus viriditarsis*) and E) a amycterine ground weevil (*Gagatophorus draco*). Bottom: F) A photograph of the real insect specimens of the 3D models captured.

Figure 8 shows high-resolution natural-colour 3D models of insects ranging from 3 mm to 30 mm in length. These 3D insect models are also available for interactive viewing at [46] and can be downloaded at [47]–[54]. The smallest of these—the 3 mm granary weevil—proved challenging to resolve due to an out-of-focus problem when its images were captured at 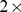 magnification. The 3D model of granary weevil was obtained from images captured in macro-mode, while 3D models of larger insects were obtained from images captured in normal-mode. The 3D visualisation of insect models is based on the open-source X3DOM framework [55] which uses WebGL for plug-in-less display within a web browser (such as Firefox and Chrome). The file size of models, including 3D mesh and texture, depends on the desired visualisation quality and the complexity of the geometry and colour of the actual specimen. For the 3D models shown at [46], the file size ranges from 5 to 24 megabytes, with number of vertices from 80,000 to 130,000 and texture resolution from 4 to 16 megapixels.

**Figure 9:**
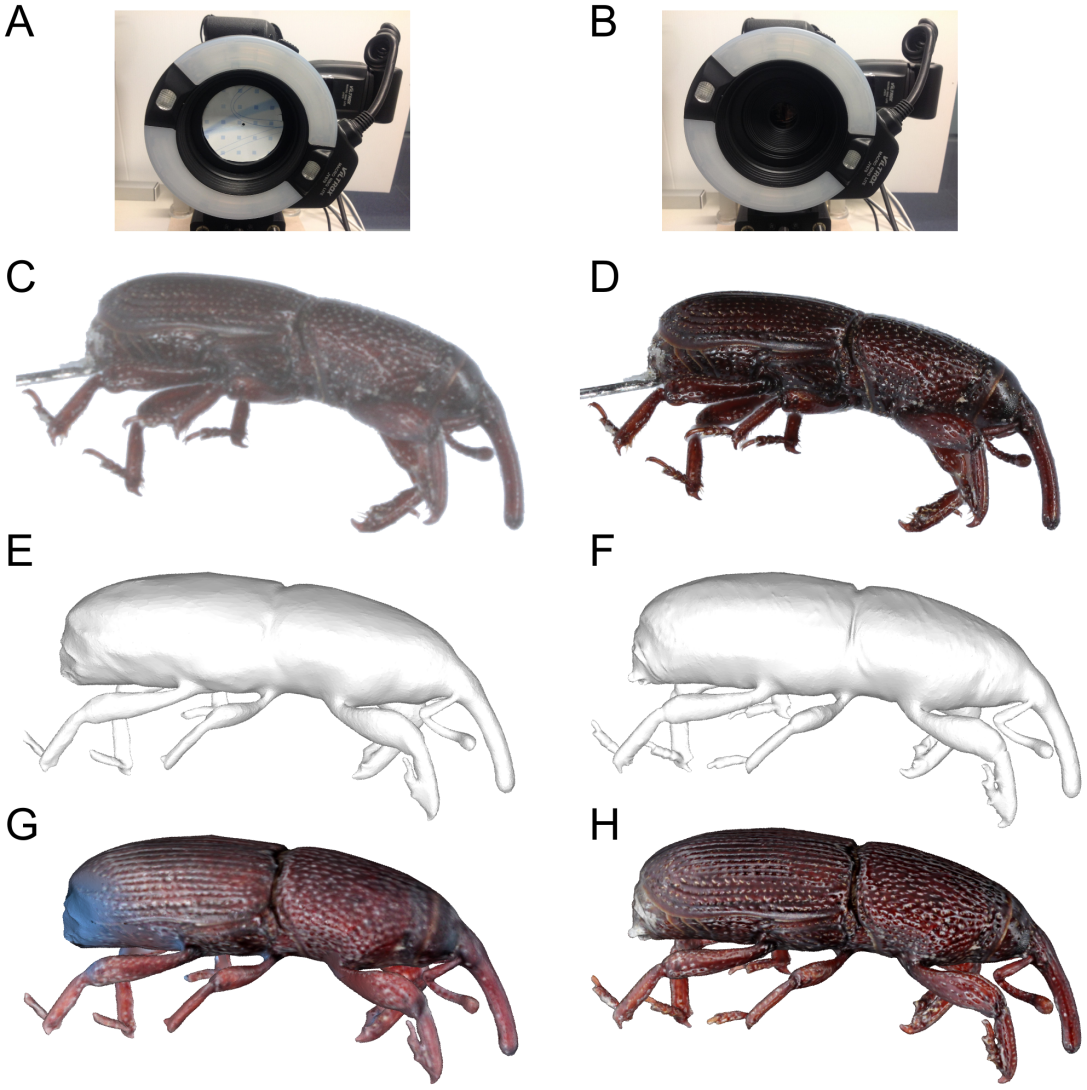
Comparison of natural-colour 3D reconstructions using (A) a small aperture and (B) a F/8 aperture with multi-focus image stacking. A) shows an extra mask with a 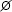
2 mm hole put in front of the lens to extend depth of focus as compared to B) an F/8 lens aperture. C) the resulting images captured at the same angle by small aperture. D) multi-focus image stacking from 31 partial-focus images captured at distances 0.25 mm apart. E)-H) show screen shots of resulting 3D models without and with texture colour.

Figure 9 illustrates the effectiveness of macro-mode image acquisition as compared to normal-mode image acquisition when applied to very small insects such as the granary-weevil. A Canon EF-65 mm macro lens was employed in both cases. In normal-mode, a stencil with a 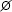
2 mm hole had to be attached immediately in front of the camera (Figure 9A) to reduce the effective aperture and increase the depth of focus. In both cases a flash was used to mitigate the effects of wobble due to the camera shutter movement. With a flash, the exposure time of an image is effectively the very short duration of the flash when it triggers, and therefore it minimizes any motion blur. Flash energy in macro mode was 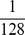 of full power and in normal-mode (for the 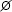
2 mm aperture) it was 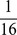 of full power. The results shown in Figure 9 clearly illustrate the improvements of macro-mode. The macro-mode model was reconstructed with multi-focus stacking of 31 images from each view, each captured with an F/8 lens aperture at increments of 0.25 mm along the specimen-camera axis.

**Figure 10:**
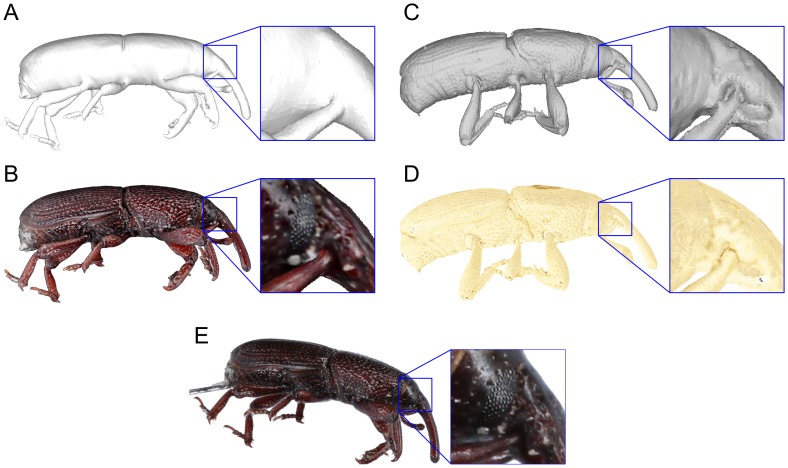
Comparison of a natural-colour 3D model, a Micro CT reconstruction and 2D image at a similar angle. The surface geometry of the natural-colour 3D model (A) is less detailed than the Micro CT model (C) and missed concavities such as the antenna socket shown in the enlarged inset of C. However, the natural-colour 3D model can capture useful surface information such as the compound eye in the enlarged insect of B. False-colour Micro CT model (D) and a 2D image (E) are shown for comparison.

Figure 10 provides a qualitative comparison of a natural-colour 3D model obtained using our system and a Micro CT model of a different specimen of the same species. While the 5.7
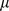
m resolution Micro CT clearly captures more details of the surface geometry than our optical approach (including the missing antenna socket in inset A), there are features that it cannot resolve at these resolutions because they are to do with variation in the colour of the specimen (e.g., the compound eye in inset B). One option could be to develop ways to combine the strengths of both approaches: fertile ground for further research.

**Figure 11:**
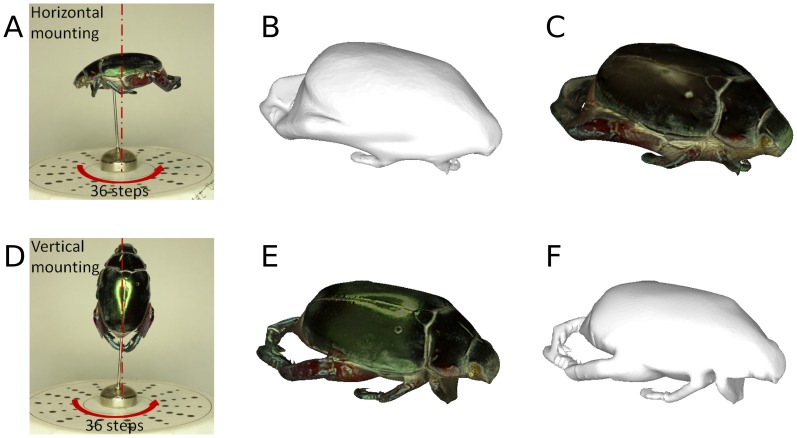
The impact of mounting orientation on reconstruction quality. Traditional horizontal mounting (A–C) produces inferior results to vertical mounting (D–F) for this specimen.

**Figure 12:**
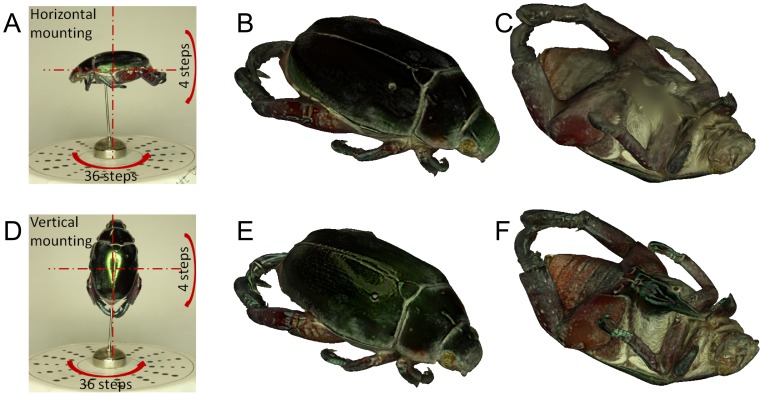
Impacts of mounting orientation and tilt on reconstruction quality. While additional images at tilting angles of 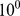, 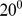, 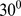 and 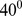 improve reconstruction quality in both horizontal and vertical mounting (in comparison with Figure 11), vertical mounting leads to sharper model with more vivid colours and textures.

By convention, insect specimens are often mounted horizontally. However this mounting orientation may not be ideal for 3D reconstruction. To investigate the effect of mounting orientation on reconstruction quality, we acquired images of a specimen mounted horizontally, then vertically (Figure 11). For the structure of that particular specimen, vertical mounting gave markedly better reconstruction of both geometry and colour, avoiding occlusions and capturing texture in more detail. Increasing the number and variety of poses by acquiring images at different tilt angles improved the reconstructions of both vertically and horizontally mounted insects (Figure 12). Even in this case, vertical mounting afforded more detail in geometry and colour. We therefore note that the best mounting orientation is specimen dependent: visual hull reconstruction of geometry improves the more surface normals are captured in silhouette, while colour and texture improve the more surface normals are captured parallel to the camera viewing axis.

**Figure 13:**
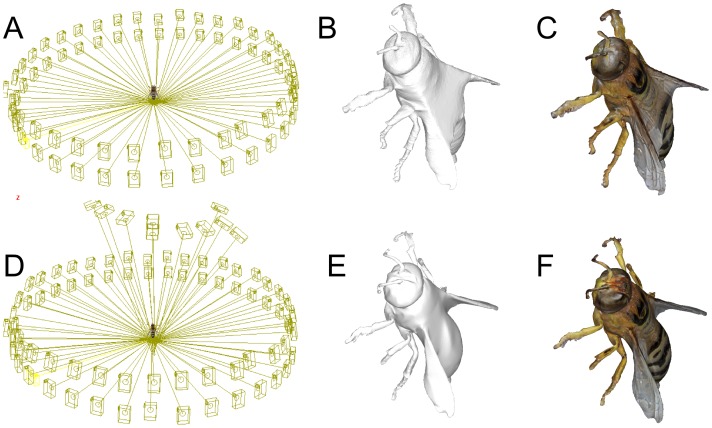
Additional camera poses can improve wing reconstruction. A) A typical set of camera poses cannot resolve the occlusion created by the wings of this insect, leading to inaccurate reconstruction between its wings (B–C). D) Additional images taken from camera poses looking along the insect body and wing surfaces dramatically improves reconstruction accuracy (E–F).

Further surface geometry issues arise as the structures of specimens become more complex. Wings, for example, can be especially challenging as shown in Figure 13(A–C) where self-occlusion causes poor reconstruction of the wings. Fortunately, additional informative views can be obtained to alleviate this problem (Figure 13D–F). Ideally, some of these additional views will be captured tangentially to the wing surface to ensure the reconstructed wings have the correct thickness.

**Figure 14:**
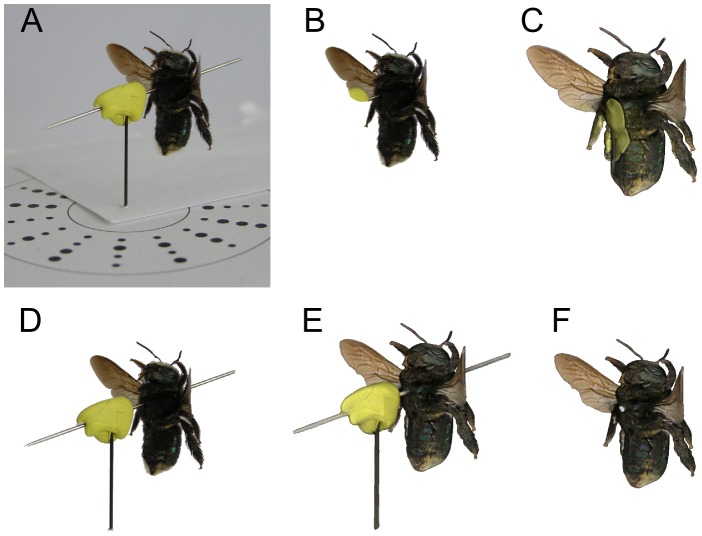
Two methods to deal with an insect whose pin cannot be removed. A) The raw image shows the pinned specimen attached to a second vertical pin so the long-axis of the insect is vertical. B) An image of the specimen after all other parts of the image are masked to some extent. C) Ventral view of the 3D reconstruction from masked images shows a splotch of contaminated texture colour. D) An image of the specimen and pins retained. E) 3D reconstruction of insect and pins. F) Ventral view of E with pins edited out of the 3D model.

We explored ways to achieve an informative mounting orientation even when the specimen cannot be re-pinned (e.g., when the specimen is too precious to handle, or the pin too firmly embedded to remove without certain damage). Previously, we mentioned that vertical orientation provides better quality than the horizontal orientation. However, repinning the specimen to have a vertical orientation causes damage, while keeping the horizontal orientation produces a lower-quality 3D model. To avoid this trade-off, the normally-pinned insect can be attached to a second pin (in this case using yellow Blu-Tack) so that the specimen is rotated on its long axis (Figure 14A). Then, the pins and the Blu-Tack need to be removed digitally to produce a clean final 3D model of the specimen. There are two methods to do this. The first method involves editing the Blu-Tack and mounting pins out of the set of 2D images (Figure 14B) during background removal prior to reconstruction. However, this method does not work well with image views where the pins and Blu-Tack occlude parts of the insect and the resulting reconstruction shows contaminated texture colour (Figure 14C). The second method is to keep the pins and Blu-Tack with the specimen during 3D reconstruction (Figure 14D and E) *then* remove them from the 3D model using a mesh editor. Overall, this second strategy produces the better result (Figure 14F).

In this paper, we have shown that high resolution, natural-colour 3D digitization system for insects and other small specimens can be implemented using readily available components with hardware and software cost under AUD8000. As well as being cost effective, the system produces digital 3D models that are fairly efficient in terms of the ratio of information to data. The file size of the 3D granary weevil model shown in Figure 9H is around 10 megabytes. It was reconstructed from 18 megapixel 2D JPEG images (2–4 megabytes/image) taken at 144 different angles and 31 different distances creating 10–17 gigabytes of 2D image data in all for a single specimen. By stacking each set of 31 multi-focus images into a single in-focus one, the image data is reduced approximately 20 times. By transforming this 2D data into a 3D model, the system further achieves a 30∶1 compression of data. This level of compression enables useful information about the specimen to be exchanged via email, presented in web pages and embedded in 3D PDF documents.

This work raises a number of research challenges and opportunities for further improvement, including:

- Eliminating the need for the printed mat: 3DSOM requires this mat to estimate the camera pose of individual images. We have reached the lower size limit of what we can straightforwardly print and attach to specimens. Furthermore, the range of poses is limited to those in which the mat is viewable. There are reconstruction methods that do not need this kind of pattern to estimate camera pose (e.g., [56], [57]), relying instead on feature matching and bundle adjustment. However, the accuracy of these estimates depend strongly on the geometry of the specimen and other objects captured in the images.
- Detailed features, such as hairs and surface roughness, demand higher 2D image and 3D model resolution and a concomitant increase in the memory and computation needed to store and visualize the model. Our strategy is to leverage the high resolution 2D image corresponding to a particular pose of interest, reminiscent of the approach used in [29].
- Concave surfaces: current photo-consistency based methods to resolve concavities can be challenged by the specular reflective properties of many insects.
- Transparent wings and membranes pose challenges for acquisition, reconstruction, and for representation and rendering of the resulting 3D model.
- View- and lighting-dependent appearance such as iridescence or sub-surface light scattering is also difficult to capture, represent and render.
- 3D annotation standards, strategies and software are not yet as developed as 2D approaches. The ability to augment 3D models with additional information is important for taxonomy and other scientific ends, as well as engaging a broader range of end users.

Despite these future challenges, we believe that the proof-of-concept prototype presented in this paper demonstrates that natural-colour 3D model digitization is feasible and affordable enough for insect collections to implement and apply right now.

An initial investigation of the usefulness of 3D insect models, as described in Supplementary Information S1, showed that the quality of 3D insect models were good enough to provide sufficient information for species identification, and allow for easier specimen examination than the actual specimen being viewed under a microscope.

The specific usage scenarios for wider communities such as quarantine officer or educator. A quarantine officer can use 3D models of invasive insects while on duty to improve the speed and the accuracy of identification process. The challenges and possible solutions by using 3D models in quarantine control have been discussed in [58]. For educators, 3D models of insects can be used as rich education materials, allowing students to interact with insects without the need to access to fragile specimens.

## Supporting Information

Supplementary Information S1 **Supporting information, figures, and table.**
(PDF)
