# Supplementary material for: Capturing Natural-Colour 3D Models of Insects for Species Discovery and Diagnostics
Source: PLoS One. 2014 Apr 23;9(4):e94346. doi: 10.1371/journal.pone.0094346 (PMC3997343; doi:10.1371/journal.pone.0094346)
Supplement: Supplementary Information S1 — Supporting information, figures, and table. (PDF) [file pone.0094346.s001.pdf]

Supplementary Information S1

Traditional insect mounting

Figure 1 shows insects mounted horizontally with a pin going through the body from the back. This mounting technique gives a strong hold on the insect body and facilitate specimen handling but provides limited access for 3D scanning.

Image processing and 3D reconstruction software

Software used for image processing and 3D reconstruction are:

- Helicon Focus [1] for multi-focus image stacking to extend depth of field. There are alternative open-source software such as CombineZP [2] and Huggins & Enfuse [3]. However we found Helicon easier to use and able to exploit multi-core processing.

- 3DSOM^{TM} software [4] for 3D reconstruction from multiple view images based on visual hull technique. The 3D reconstruction pipeline used by 3DSOM^{TM} is described in [5]. There are other 3D reconstruction software including commercial software Agisoft Photo Scan [6]; and open-source softwares Bundler [7] and Patch-based Multi-view Stereo [7, 8], and visual hull mesh software [9].

These two off-the-shelf software packages, together, cost around AUD1700 based on 2013 prices.

Image acquisition equipment

We use the following off-the-shelf components:

- A GigaPan^{TM} Panorama Robot EPIC 100 [10]. Normally this device is used to control the tilt and pan of a camera to capture panorama images. In this project, we turn it side-ways and use it as a two-axis turntable to control the tilt and pan of the specimen to be imaged. The GigaPan^{TM} Panorama Robot connects to a camera via a cable and triggers a camera to capture one or more images per rotation angle.

- A Canon^{TM} 600D camera or better. It provides low-noise, high resolution images (18MP). It is highly customizable with several ports for external trigger input, flash trigger output, and a USB port for remote tethering. One problem with many DSLR cameras is that they rely on a mechanical shutter which can cause mechanical vibration and image blur. The image blur due to mechanical shutter vibration can be alleviated by using a camera flash triggered between shutter movement.

- A Canon^{TM} EF 100mm macro lens for normal-mode and Canon^{TM} MP-65mm for macro mode. The EF-100mm lens provides magnification of 1× or smaller, while the MP-E 65mm lens provides magnification from 1× to 5×. These macro lenses also have much less optical distortion than some other lenses. A low-cost solution to make normal lens achieve higher magnification is to add a macro extension tube between the camera and the lens. However this often increases optical distortions [11]. The depth of field of MP-65mm lens as function of magnification and aperture can be obtained from its user manual.

- A Viltrox^{TM} JY-670 macro ring flash [12]. This flash provides illumination and reduces exposure time. Short exposure time is critical for macro photography to reduce image blurring due to vibration from the camera's mechanical shutter. Additionally, a Tronix SpeedFire flash power supply [13] is used for fast flash charging.

- A StackShot™ macro-rail [14]. This device enables us to capture high-magnification partially-focused images at predetermined depth intervals with high position accuracy. This macro-rail can run automatically to capture images along a single direction. The process of movement and image acquisition using macro-rail starts with a press of "Up" button on the rail's control panel. To synchronize StackShot™ macro-rail and GigaPan™ robot, we built a circuit interface, shown in Figure 2, to convert the robot's trigger signal to a press-button effect. The step size of the rail is set to be approximately 70% of the depth of field of the macro lens to allow for adjacent overlapping required for stacking multi-focus images.
- Two 1mW laser pointers [15] for specimen alignment. The laser beams are used to locate the specimen at the center of rotation of the two-axis turntable so that the specimen stays at the same position while being rotated and imaged.
- Aluminium frames [16] to hold the turntable, laser pointers, camera, and macro-rails in position.
- Button magnets [17], plastic spacers [18], epoxy glue and pins to mount insect specimens.

The system does not need any special software for acquiring images. The EOS Utility software accompanying the camera is needed to remote-tether the camera and transfer images to a computer during image acquisition. Based on 2013 prices, we estimate the total hardware to cost around AUD3200 to AUD5600. Examples of actual acquisition setups are shown in Figure 3.

Estimation of processing time

The reconstruction process described in this paper is a proof of concept and not yet optimized for speed and for large-scale digitization. A time estimation for each stage of the 3D reconstruction procedure for the insects shown in paper is provided in Table 1. The longhorn beetle, Christmas beetle and Amycterine ground weevil take relatively similar amount of time to obtain a 3D model. The sand wasp has increased reconstruction time due to the extra time required to manually correct the errors of background removal around the wings, and to refine camera pose during reconstruction (hairs significantly increase the time for pose refinement). The granary weevil, due to its small size, takes much more time to mount using a microscope, acquire multi-focus images and perform 3D reconstruction. It is often the case that the reconstruction stage is repeated several times to find and fix errors in background removal, or to iterate the refinement process until the result is acceptable. Therefore, the times provided in Table 1 should only be considered as indicative.

Informal feedback on using a 3D insect model for species identification

One possible use case for 3D insect models is species identification. In drafting this paper, we needed to fully identify the Christmas beetle. We asked CSIRO entomologist Mr Tom Weir to try to do so from the photograph, the 3D model, and finally the actual specimen. In addition to identifying the specimen as a male *Anoplognathus viriditarsus* from the 3D model alone, Mr Weir provided feedback which we summarize as:

- The single top-view photograph does not capture the key features needed to identify the species, just enough information to determine its genus. The features are located at different parts of the insects such as abdomen, head, mouth, claws, rear end, etc. Multiple images captured at particular angles are required to show all these features.

- The 3D model provides useful extra information and allows views from any angle. There are still missing features such as the mouth area and hairy surface on the head/nose due to resolution of geometries and texture colours. However the available features are roughly enough to identify its species. Examination and species identification would be facilitated if the texture information was provided more clearly, or high-resolution images of the features were attached to the model.
- The actual specimen obviously provides all the information needed but needs examination under a microscope for some features such as the mouth area and hair surface on the head. However, out-of-focus effect and other physical restrictions makes the use of microscope to view the actual specimen more cumbersome than to view the 3D model of specimen on a mobile device (such as iPad).
- Identification involves matching the given specimen to its corresponding specimen in the Australian National Insect Collection *and* comparing it to closely related species.
- For identification purposes, the natural-colour 3D model is much more helpful than the Micro CT 3D model. The better geometry accuracy of the Micro CT model does not assist in this instance.
- Higher resolution of the natural-colour 3D model is desirable to provide details of key identification features. This is particularly important for features, such as hairs, that never get included in the 3D models. One solution would be to attach high resolution photographs to locations of the key features.
- To make the 3D model useful for species identification, it is important to know in advance all key features and the identification procedure and level. Ideally, this information would be arranged in a way that directly supports fast, correct identification. 3D visualization and annotation could make the identification procedure more obvious that the instructions currently provided in text and 2D illustrations.

References

1. HeliconSoft (2013). Helicon Focus. URL `http://www.heliconsoft.com/heliconsoft-products/helicon-focus/`.
2. Hadley A (2010). CombineZP. URL `http://www.hadleyweb.pwp.blueyonder.co.uk/CZP/News.htm`.
3. D'Angelo P (2013). Hugin & Enfuse. URL `http://hugin.sourceforge.net/tutorials/index.shtml`.
4. Creative Dimension Software Ltd (2013). 3DSOM. URL `http://www.3dsom.com`.
5. Baumberg A, Lyons A, Taylor R (2005) 3D S.O.M.A commercial software solution to 3D scanning. Graphical Models 67: 476–495.
6. Agisoft (2013). Agisoft PhotoScan. URL `http://www.agisoft.ru/`.
7. Snavely N (2010). Bundler: Structure from Motion (SfM) for Unordered Image Collections. URL `http://www.cs.cornell.edu/~snavely/bundler/`.
8. Furukawa Y (2010). Patch-based Multi-view Stereo Software. URL `http://www.di.ens.fr/pmvs/`.

9. Forbes K (2009). Visual Hulls from Uncalibrated Snapshots. URL http://www.dip.ee.uct.ac.za/~kforbes/DoubleMirror/DoubleMirror.html.

10. GigaPan Systems (2013). The GigaPan Store - Products, software and accessories to create gigapixel panoramas. URL http://gigapan.com/cms/shop/store.

11. McHugh S (2013). Macro extension tubes and close-up lenses. URL http://www.cambridgeincolour.com/tutorials/macro-extension-tubes-closeup.htm.

12. JVC Technology (2013). Viltrox JY-670 macro ring lite. URL http://www.viltrox.com/en/Products/flashlight/2012/1121/2.html.

13. Innovatronix Inc (2013). Tronix SpeedFire. URL http://www.innovatronix.com/index.php/products/item/35-tronix-speedfire.

14. Cognisys Inc (2013). StackShot - Focus stacking macro rail. URL http://www.cognisys-inc.com/stackshot/stackshot.php.

15. Dick-Smith (2013). Keychain 1mW Laser Pointer. URL http://www.dicksmith.com.au/office-accessories/keychain-1mw-laser-pointer-dsau-xp0671.

16. Bunnings (2013). Castor Cup Round Tic 48mm Clear Plastic 25685. URL http://www.bunnings.com.au/castor-cup-round-tic-48mm-clear-plastic-25685_p4050109.

17. Frenergy Magnets (2013). Neodymium Disc Magnet 10mm dia. x 3mm height N50. URL http://www.frenergy.com.au/products/Disc-10mm-dia.-x-3mm-height-N50.html.

18. Bunnings (2013). Metalmate RCR 25.4 x 25.4 x 1.2mm 1m Aluminium Square Box Tube. URL http://www.bunnings.com.au/metalmate-rcr-25-4-x-25-4-x-1-2mm-1m-aluminium-square-box-tube_p1067815.

19. Nguyen C, Adcock M, Lovell D, Mantle B, La Salle J (2014). Insect collections going 3D. doi:10.4225/08/531FE0104C11A. URL http://dx.doi.org/10.4225/08/531FE0104C11A. Viewable at http://www.youtube.com/watch?v=THvfu6shJjg&list=UUak3NZxjNnWip327vYL8RLA or http://publish.viostream.com/play/xbz189zsuhn8.

Figure Legends

Tables

Table 1. Time consumption estimation

Insect	Mounting (min)	Acquisition (min)	Reconstruction (min)
Longhorn beetle, Christmas beetle and ground weevil	5	15	60
Sand Wasp	5	15	120
Granary Weevil	30	180	120

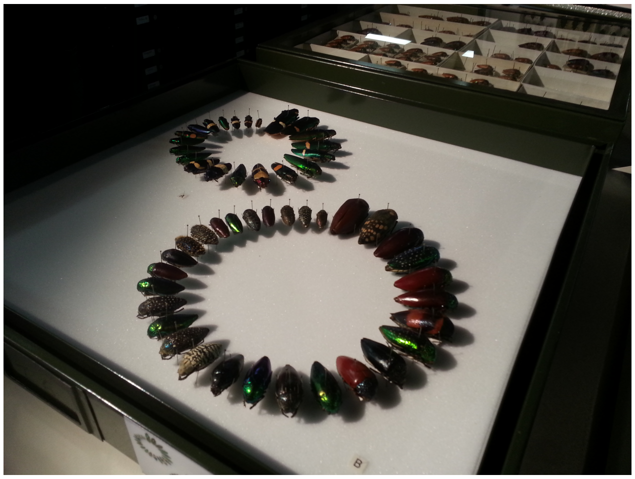

Figure 1. Traditional insect mounting. A metal pin pierces the body of an insect from the back providing stronghold to the specimen. However this mounting can be inconvenient for 3D scanning due to the speciment's horizontal body axis.

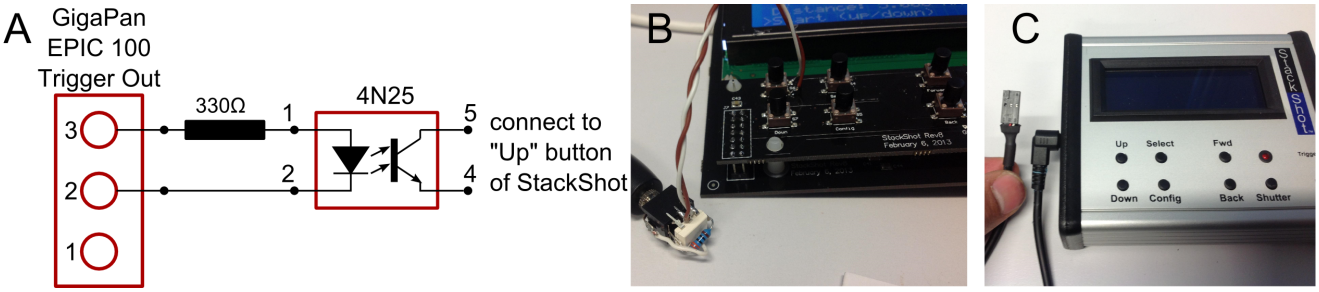

Figure 2. Modification of StackShot^{TM} macro-rail's control box to accept external trigger. A) Circuit diagram to provide interface between trigger output of GigaPan^{TM} robot and StackShot^{TM} control box. The resistor and the opto-coupler convert the trigger signal to a "press button" action. B) the resistor and opto-coupler are soldered to a connector which can be mounted to the case of the control box. C) Finished the control box with the extra input connector on the left. Note that the connector pins of GigaPan^{TM} trigger cable have to be swapped such that red cable goes to position 2 and white cable to position 3.

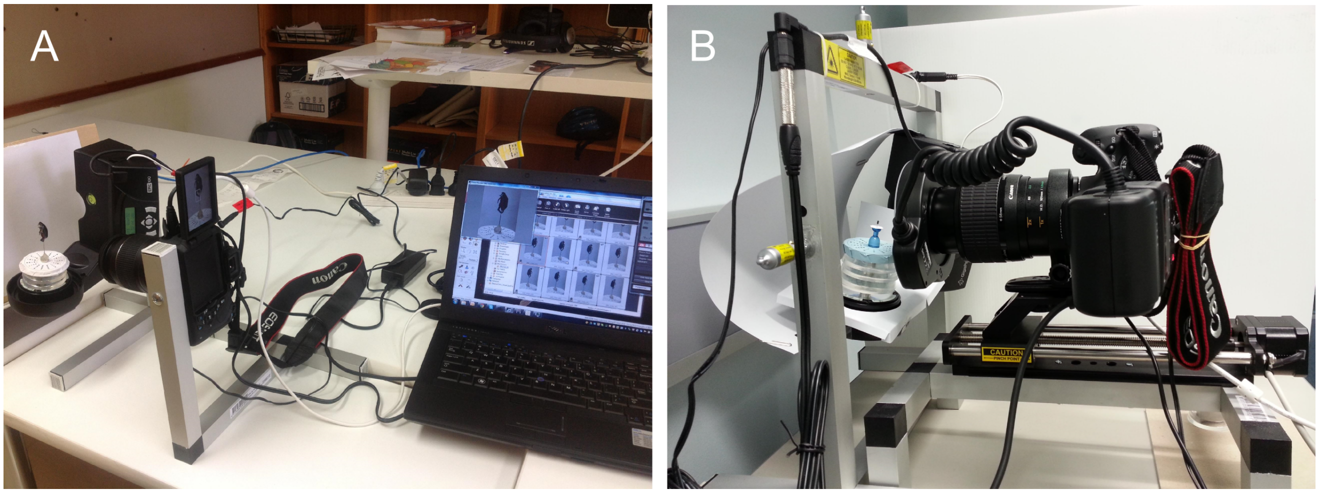

Figure 3. Examples of actual set-up in normal-mode (A) and macro-mode (B). The GigaPan^{TM} Panorama Robot is mounted sideways to act as a two-axis turntable. The camera is turned 90^0 around optical axis so that it can capture images that better fit the specimen and the mat target. Video of the system (B) at work are available at [19].
